# Expectations and needs of socially vulnerable patients for navigational support of primary health care services

**DOI:** 10.1186/s12913-021-06811-8

**Published:** 2021-09-22

**Authors:** Carine Sandrine Ngo Bikoko Piemeu, Christine Loignon, Émilie Dionne, Andrée-Anne Paré-Plante, Jeannie Haggerty, Mylaine Breton

**Affiliations:** 1 Department of Community Health Sciences, Université de Sherbrooke, Longueuil, Canada; 2 Centre de Recherche-Hôpital Charles-Le Moyne – Saguenay Lac-St-Jean sur les Innovations en Santé, Longueuil Campus, 150 Place Charles-Le Moyne, Office 200, Longueuil, J4K0A8 Canada; 3 Department of Family Medicine and Emergency Medicine, Université de Sherbrooke, Longueuil, Canada; 4 VITAM – Centre de Recherche en Santé Durable, Québec, Canada; Department of Sociology, Université Laval, Québec, Canada; 5 Charles-Lemoyne University Medicine Group, Saint-Lambert, Canada; 6 Department of Family Medicine, McGill University, Montréal, Canada

**Keywords:** Navigational support, Expectations and needs, Vulnerable patients, Primary healthcare

## Abstract

**Background:**

Primary healthcare is the main entry to the health care system for most of the population. In 2008, it was estimated that about 26% of the population in Quebec (Canada) did not have a regular family physician. In early 2017, about 10 years after the introduction of a centralized waiting list for patients without a family physician, Québec had 25% of its population without a family physician and nearly 33% of these or 540,000, many of whom were socially vulnerable (SV), remained registered on the list. SV patients often have more health problems. They also face access inequities or may lack the skills needed to navigate a constantly evolving and complex healthcare system. Navigation interventions show promise for improving access to primary health care for SV patients. This study aimed to describe and understand the expectations and needs of SV patients.

**Methods:**

A descriptive qualitative study rooted in a participatory study on navigation interventions implemented in Montérégie (Quebec) addressed to SV patients. Semi-structured individual face-to-face and telephone interviews were conducted with patients recruited in three primary health care clinics, some of whom received the navigation intervention. A thematic analysis was performed using NVivo 11 software.

**Results:**

Sixteen patients living in socially deprived contexts agreed to participate in this qualitative study. Three main expectations and needs of patients for navigation interventions were identified: communication expectations (support to understand providers and to be understood by them, discuss about medical visit, and bridge the communication cap between patients and PHC providers); relational expectations regarding emotional or psychosocial support; and pragmatic expectations (information on available resources, information about the clinic, and physical support to navigate the health care system).

**Conclusions:**

Our study contributes to the literature by identifying expectations and needs specified to SV patients accessing primary health care services, that relate to navigation interventions. This information can be used by decision makers for navigation interventions design and inform health care organizational policies.

## Background

### Access to primary healthcare

Primary healthcare (PHC) is the main entry to the healthcare system for most of the population, even for specialized care. In Canada, in 2008, approximately 16% of the population reported not having a regular family physician, with rates as high as 26% in the province of Québec [1]. These patients without a family doctor often do not know where to go to get the healthcare and services they need. This can lead to inappropriate usage of health services and often end up in unneeded use of the emergency room. As a result, health services costs increase.

To address this problem of patients without a family physician in PHC, in 2008, seven Canadian provinces, including Quebec, implemented centralized waiting lists [2] on which patients register and that centralize requests for a primary care provider in a given territory and match patients according to urgency of medical need and availability of family physician [3]. In Quebec’s universal public healthcare system, only patients covered by the province’s public health insurance plan can be registered on waiting lists [4]. Once on a list, they are in a queue and will be contacted by the next available.

Formal registration of patients to family physicians is relatively new in Quebec; it began with the implementation of Family Medicine Group (FMGs) model and that was extended to all healthcare settings. FMGs are PHC clinics (typically referred to as ‘clinics’) and consist of a group of physicians working in close collaboration with nurses to provide services to patients in one clinic [5]. There are three types (FMG, University FMG, and super-clinics). Only family physicians working in FMGs can take patients who are on waiting lists.

In Québec, in early 2017, nearly 10 years after the implementation of this list approximately 25% of the population was without a family physician [6] and nearly 33% of these, or 540,000, remained registered on the list many of whom were socially vulnerable (SV) [2, 7]. SV individuals have demographic, social, geographical, or economic characteristics that often impede or compromise their access to comprehensive, quality primary healthcare and services (Haggerty J, et al: Development of an index of social vulnerability that predicts negative healthcare events: A proposed tool toward healthcare equity in primary care Montreal, In progress). Yet, they often have a greater need for healthcare services than non-SV individuals. Their needs may be more complex in that these individuals may have several chronic illnesses, mental health issues, medication-related issues, or problems related to communication with the medical team. They are also more likely to require healthcare services from a variety of professionals and organizations (e.g., medical specialists) and social services. Thus, for SV patients, it can be difficult to navigate the complex and constantly evolving healthcare system [8–10].

### Healthcare system navigation services

Navigation interventions appear promising to improve access to PHC for various vulnerable sub-population groups, including SV ones [11], and to reduce socioeconomic disparities in healthcare [12]. Navigation interventions are defined as the assessment and alleviation of barriers to adequate health care by a trained lay person [12]. Studies have shown that navigation interventions increase the likelihood that low-income patients have a family physician [13], contribute to reduced emergency room use by SV patients [14] and associated costs [15], and facilitate the work of PHC teams by improving communication between the patient and the medical team [14]. Moreover, research has shown that the emotional support provided by these interventions is beneficial to SV patients [12, 15].

Navigation services can be grouped into two categories. First, pragmatic supports including, for example, assistance with referrals to support groups and counselling services [16, 17], transportation [16, 17], information on existing resources [12, 18, 19], planning appointments [16, 17, 19, 20], completing forms [21], and care coordination [21]. Second, relational, interpersonal or emotional support, including, for example, assistance with decision-making [21], follow-up care [15], and response to emotional distress (e.g., listening supportively, providing comfort) [12, 20]. Both categories are delivered by a navigator with or without professional or clinical expertise (e.g., patients, nurses, social workers, health educators, community health workers, medical assistants, or volunteers) [15].

While it is clear that SV patients appreciate navigation interventions [12, 16] and expect that they provide emotional comfort, and help making decisions, communicating health problems to doctors, and overcoming logistical barriers, such as insurance, transportation, and scheduling appointments [12, 22], little is known about which type of navigation interventions model is best suited for particular situations [23, 24]. A better understanding of the expectations and needs of SV patients could help decision-makers design, implement, and improve PHC navigation interventions [15]. The purpose of our study was, therefore, to describe and better understand the expectations and needs of SV patients regarding navigation interventions. The specific objectives of this study were: 1) to explore the expectations and needs regarding navigation interventions according to SV patients who had received the intervention as well as those who had not; and 2) to describe the appreciation of SV patients who had received the navigation intervention.

## Methods

### Context of the study

Whithin the context of an international participatory research program (IMPACT: Innovative Models Promoting Access to Care Transformation) [25–28], a study on the navigation intervention (Titled “Patient Welcome Service” (PWS)) describe briefly below, was conducted in the Montérégie of Quebec. Our study was conducted in the context of this PWS which was codesigned, implemented and evaluated in partnership with local healthcare organizations and clinicians, to enhance the likelihood of establishing an enduring relationship between SV patients and the FMGs where their family physicians work (Haggerty J, et al: Harnessing the power and passion of lay volunteer navigators trained to improve access to care, In progress). A full description of PWS can be find in other publications (Haggery J, et al: Harnessing the power and passion of lay volunteer trainers to increase access to care, In progress).

Our study aimed, through a descriptive qualitative approach, to meet the above-mentioned objectives. Our study did not evaluate the PWS as this was the focus of a different study that will be published elsewhere.

### Study design

We use a descriptive qualitative study [29], to describe and understand the expectations and needs of SV patients regarding navigation interventions.

### Participants

Sixteen patients were recruited for this study - 9 patients who received the intervention (referred to as intervention patient) and 7 who did not receive it (referred to as non-intervention patient). We used a mixed sampling strategy (purposeful sampling (criteria are listed below), and snowballing) to select participants [30, 31]. We sought to reach variation primarily in terms of patients’ experience of access to care (i.e., types of difficulties encountered) and degree of social vulnerability, and their background in terms of gender and age. Participants were recruited either in person by their family physician or the social worker of the FMG, or over the phone by a senior research assistant.

To be eligible for the large study on the PWS, participants had to have been recently enrolled with a family physician, have at least one socio-material vulnerability criterion (low income, social assistance, isolation (little social support), low education (secondary 5 or less), low health literacy (patient has major difficulties navigating the health system, communicating their problems, etc.), be unemployed, be in single parent family) [10, 32–34], speak English or French, and be at least 18 years old. Participation in the study was voluntary.

### Description of navigator intervention

For intervention patients: Volunteer navigators were recruited (following complete background checks), signed confidentiality agreements, and were trained to connect with patients by telephone and to provide them help to communicate with their physician or to provide pragmatic supports. Communication help included, for example, suggesting the patient bring a written list of questions to their appointment, so as not to forget any, or suggesting they ask their physician for clarifications, as needed, during their appointment. Pragmatic supports included help acquiring home care or an appointment with a medical specialist, help to prepare for an initial medical appointment. Specific examples are providing the patient with FMG addresses, opening hours, and services; a list of what to bring to the appointment, and help searching for and acquiring information about community resources (e.g., telephone numbers, addresses, contacts for scheduling or canceling appointments or obtaining service referrals).

### Data collection

Data collection took place between January 2017 and August 2018. Semi-structured individual interviews with participants were conducted by telephone or in-person at their FMG, as per their preference. For the interviews in this study, each participant provided free and informed consent. To ensure that participants provided informed consent, at the beginning of each interview, the interviewer made sure the participant had read and understood the consent form, reviewing it providing explanations and answering questions, as needed, and explaining how their confidentiality and privacy would be ensured. The interviews ranged in length from 25 to 60 min and were conducted by female research assistants trained in qualitative research and interviewing (one senior qualitative PhD researcher (KC), with a special interest in access to healthcare services for the vulnerable and one master student (CSNBP), trained by the senior qualitative PhD researcher). We used an audio recording to collect the data. The interviews, transcripts and analyses were any conducted primarily in French. Two interview guides were developed by the qualitative researchers on our team (ED, CL, MB, AAP, JH, CSNBP): one for those who had received the intervention and one for those who had not. They were based on a literature review of primary care access issues, social vulnerability, and navigation interventions [26–28, 35] and piloted with SV patients not participating in this study. Both included questions related to access to healthcare and social services, and expectations and needs for navigation interventions. Each participant received $15 to cover transportation costs.

The socio-demographic characteristics of participants are summarised in Table 1.

**Table 1:** Socio-demographic characteristics of participants (*n* = 16)

	Total	
	N	%
**Total Number of participants**	16	100%
**Characteristics**		
**Age in years, mean (SD), median (range)**	49.4 (13.8)	46 (23–78)
**Sex (n, %)**		
Female	9	56%
Male	7	44%
**Race/ethnicity (n, %)**		
Caucasian	15	94%
Black/African-American	1	6%
**Main language (n, %)**		
French	14	87%
Other	2	13%
**Country of birth (n, %)**		
Canada	13	81%
Outside of Canada	3	19%
**Insurance (n, %)**		
Public	13	81%
Public and private	3	19%
**Length of time with family physician (n, %)**		
1 to 6 months	8	50%
7 to 12 months	4	25%
13 to 18 months	0	0%
19 to 24 months	4	25%
**Civil status (n, %)**		
Single	5	31%
In a couple/married	10	63%
Separated/divorced/widowed	0	0%
Unknown	1	6%
**Family status (n, %)**		
Single parent family	5	31%
Non-single parent family	11	69%
**Schooling (n, %)**		
≤ Secondary 4 (10th grade)	5	31%
Secondary 5 completed (11th grade)	5	31%
University	6	38%
**Employment (n, %)**		
Employed	7	44%
Unemployed	6	37%
Not available	3	19%
**Number of chronic diseases (n, %)**		
0	5	31%
1	9	56%
3 to 4	2	13%

### Data analysis

We used hybrid (deductive and inductive) thematic analysis [36] based on the two categories of navigation services identified in the literature review (described above). To organize the data and assist with the analysis, we used NVivo 11 software [37]. The first author developed an initial codebook according to the literature on the two categories of navigation services [12, 15–21] and on patient appreciation of navigation interventions [12, 16, 22], and CL and MB reviewed and approved it. CSNBP and a qualitative PhD (PA) researcher independently coded the first four interview transcripts, meeting several times to discuss and debate codes, and to refine the codebook. We used this codebook when coding subsequent transcripts, which were each coded by at least four team members. As part of this process, new codes, categories, were generated and discrepancies were resolved by team consensus. We looked for negative cases to improve credibility. We triangulated the data by analysing the data from both groups together to address objective one and separately to address objective two. We looked at the frequency with which each expectation or need and appreciation were expressed by the participants. We used constant comparison between the data and codes to improve the trustworthiness of the analysis. Interpretations were discussed and validated by the whole team during several meetings. Participant quotes included in this article were translated into English and accuracy was verified by two co-authors. We reached theoretical data saturation for the corpus of data at the twelfth interview (7 intervention and 5 non- intervention participants) but completed analysis of all 16 interviews.

## Results

Nine of the sixteen participants had low-income status (less than $24,000 CAN/year), and five had not completed high school. More than half reported living with at least one chronic disease.

### Patient’s expectations and needs of navigation interventions

Three themes were found about patient’s expectations and needs for navigation interventions: (a) communication expectations and needs; (b) relational expectations and needs and (c) pragmatic expectations and needs (see Table 2). These are discussed below, in turn.

**Table 2:** Results of thematic analysis

	Themes	Sub-themes
**Patients’ expectations and needs**	**Communication expectations and needs**	*. Needs in terms of understanding providers*
		*. Expectations in terms of making oneself understood or heard by providers*
		*. Needs to discuss about medical visit*
		*. Expectations to help bridge communication cap between patients and PHC providers*
	**Relational expectations and needs regarding e*motional or psychosocial support***	
	**Pragmatic expectations and needs**	*. Information on ressources*
		*. Information about the FMG (*e.g. *policies,*. *.. resources, or providers)*
		*. Physical support*
**Appreciation of patients who had received navigation interventions**	**Appreciated**	*. Communication support*
		*. Information about resources*
		*. Pragmatic aspect of preparing for the medical appointment*
		*. Emotional support*
		. Appreciated but unhelpful

#### Socially vulnerable patients communication expectations and needs

The theme ‘communication expectations and needs’ was found in the data of both groups of participants. This theme includes: a) needs help to understand health and social service providers; b) expectations to successfully express oneself or to make oneself heard; c) needs to have communication tips for medical visit; and d) expectation to help bridge the communication gap between patients and their PHC providers.

- Needs help to understand health and social service providers

Results suggest that SV patients need navigation interventions to provide help to understand health and social service providers. In particular, SV patients expect to be able to understand the information or instructions their family physician provides:

*“[Navigator] could also ask health questions for me when I don’t understand what [health professionals] are saying, especially when I communicate with my doctor.” [non- intervention patient 06]*

b) Expectations to successfully express oneself or to make oneself heard

Results suggest that SV patients expect navigation interventions to improve their communication with providers by, for example, helping them to improve their communication skills (e.g., how to express themselves or make particular demands, such as wanting doctors to ask more questions):

*“[navigation interventions] could also help me explain my symptoms and talk.” [intervention patient 03]*

c) Needs to discuss about medical appointment

Results suggest that SV patients expressed a need to discuss about medical appointment as a need in relation to navigation interventions. They thought that a navigator could help by calling them to talk about their medical appointment in advance and by providing communication tips, such as preparing written questions in advance:

*“Yes. [Navigator] helped me to prepare [for my initial appointment]. I remember using the pamphlet I received; I wrote down my questions.” [intervention patient 06]*

d) Expectation to help bridge the communication gap between patients and their PHC providers.

Results suggest that SV patients emphasized the expectation for the navigator be a person to whom they could turn for information (e.g., from their medical records), and who would facilitate information sharing between health professionals and patients when, for example, they felt their appointment was not sufficiently long to address all of their concerns or when their family physician was unavailable. For instance, one participant said when referring to speaking with their family physician:

*“I would like for [navigator and I] to talk together about all of my concerns, all of my problems, and for [navigator] to put together her file and to go see my family physician to explain it better. (...) [family physicians] don’t evaluate me well, they are in a hurry (...).” [non- intervention patient 02].*

#### Relational expectations and needs regarding emotional or psychological support

The second theme found in the data of both groups regarding patients’ expectations and needs for navigation interventions is ‘relational expectations and needs regarding emotional or psychological support’. Results suggest that SV patients expect or need navigation interventions to provide emotional or psychosocial support such as comfort and support in times of distress. For example, SV patients described that they often felt greatly affected by their health problem, so they expected the navigator to support them when receiving bad news. As expressed by one participant:

*“(…) I [expect navigator] to be able to provide some emotional support when I get bad medical news (…).”* [*non- intervention patient 06]*

As another example, SV patients expressed an expectation that the navigator be unrelated to the doctor, perhaps a lay person:

*“(…) [Navigator] could be someone I feel comfortable with. I would like [navigator] to be a person separate from the doctor.” [*non- intervention *patient 01]*

#### Pragmatic expectations and needs

The third theme expressed by participants of both groups is ‘pragmatic expectations and needs’ which includes: a) information on resources, b) being informed about the FMG (e.g., policies, resources, or providers), and d) physical support.

- Information on resources

The expectation of navigation interventions most often expressed by SV patients was the assistance with information on the availability and location of available resources, such as health resources and community resources. Some participants who had health problems reported the importance of the navigator advising them where to go for health and community care and services (e.g., specialists).

*“(…) [a navigator] who (...) informs me about health services as well as about social services to go to for my health problem. For example, how to get a specialist (...). If the [navigator] had been there, they would have been useful for me in this situation to give me the information (...)”* [non- intervention patient 04]

Another example is the need for resources that are accessible in terms of, for example, cost or schedule.

*“One time, I told my doctor that I needed to find a physiotherapist who is available on the weekend. I can’t find any myself. I’m often away due to my work.”* [intervention patient 09]

b) Being informed about the FMG (e.g., policies, resources, or providers)

Results suggest that SV patients expect or need navigation interventions to provide information about the FMG’s policies and procedures, such as which FMGs were currently taking patients and what services they offered:

*“(…) for [navigator] to look for resources close to home for my needs (...) I would like [navigator] to tell me which [FMG] have more appointments available (...) I don’t know the [FMG] (...) I would like that the [navigator] help me find my way through the healthcare system.”* [non- intervention patient 01]

Other patients reported expecting navigation interventions to provide information about the doctor. For example, the ones who use their phones less and spend more time with their patients:

*“It would be good to receive more information about the doctor (…) It would have helped if the doctor would have spent more time with patients and less on their phone.”* [intervention patient 09]

c) Physical support

The third sub-theme is physical support. Results suggest that SV patients expect or need the navigation interventions to provide physical support such as mobility assistance or accompaniment. For example, due to the effect of their health conditions on their mobility, some SV patients explained needing assistance travelling to and from appointments:

*“(...) I would need a [navigator] (…) to accompany me, to help me get around. For example, when I had blood pressure drops, I went to the hospital for an appointment, I stepped onto the sidewalk and then fell. The [navigator] could help me get around.”* [non- intervention patient 04]

*“(…) I have difficulty getting around because I’m weakened by my health problems (...) I would like [navigator] to accompany me in the healthcare system (...).”* [non- intervention patient 06]

Other SV patients expected the navigator to accompany them to medical appointments to explain their health problems or concerns (e.g., find the right words) or to provide them reassurance when seeing a specialist, given their worries regarding what would happen during the appointment.

*“(...) I would like [navigator] to know my needs (...) when I don’t feel comfortable asking questions about certain situations, [navigator] must be there to do it for me.” [non- intervention patient 01]*

*“The navigator would also be useful for me, for example when I have to go to see a specialist, I need someone (…) neutral to provide reassurance.” [non- intervention patient 04]*

### Patients appreciation of navigation interventions

Among those who had received the PWS, some had appreciated it. Others had appreciated it, but had not found it helpful. Patients appreciated: (a) the communication support, (b) the information provided about resources, (c) the pragmatic aspect of preparing for the medical appointment, and (d) the emotional support.

- Communication support

SV patients said that they had appreciated the PWS and, in particular, found that the navigators provided helpful tips (e.g., to prepare questions in advance) that they would not have thought of themselves:

*“[Navigator] asked me to write down all the questions I thought I might ask the doctor. (...) yes [I appreciated it], because I hadn’t thought to write down what I could talk about it with regards to my health (...) the advice and suggestions [navigator] gave were useful for my first appointment with the doctor” [intervention patient 06]*

b) Information about resources

Many SV patients appreciated receiving information (e.g., phone numbers) about resources available at the FMG or in the community that could be useful in the future:

*“I appreciated the [navigator’s] assistance (...) she gave me (...) the leaflet containing information on resources available for any future health problems.” [intervention patient 08]*

They also appreciated receiving information on how to book or cancel an appointment with their family physician or receiving other practical information such as hours of operation:

*“(...) then also to find out what to do if I want to cancel an appointment or make an emergency appointment. And also to call the [FMG] if I want to get an appointment. I found [PWS] very good” [*intervention patient 06]

c) Pragmatic aspect of preparing for a medical appointment

Many participants who received the PWS appreciated the pragmatic aspect of preparing for medical appointments. This appreciation was expressed equally by male and female participants. For example, participants expressed that they appreciated having a list of the documents they needed to bring to their appointments. They appreciated receiving tools and tips because they acted as reminders of what to bring, what to ask, and what to do following the appointment (e.g., instructions or referral procedures):

*“I appreciated the [navigator’s] guidance for the first appointment with the doctor, how to prepare, what to bring, the health problems to discuss. I appreciated it because (...) when I arrived at the family doctor’s office (…) I knew what to say to them (...).”* [intervention patient 08]

*“The call with the volunteer went well (...) yes, it helped me a lot, the information that (...) the [navigator] gave me during the call, such as to bring all my health documents (...) She told me not to forget my health insurance card, my medication list. She told me what not to forget.”* [intervention patient 01]

d) Emotional support

SV patients appreciated the emotional support the PWS provided. For example, the navigator helped them to overcome the stress associated with a medical appointment.

*"(...) [PWS] helped me in the sense that [navigator] prepared me psychologically, and morally she also prepared me (...) the service that the [navigator] gave me helped me to combat stress, for example, I was less stressed, I was safer at least.” [intervention patient 02]*

For SV patients, not knowing how long to wait for a family physician was a source of anxiety. Receiving a call from the navigator to tell them the date and time of their appointment provided them relief:

*“The phone call came to me as a relief; I no longer knew where to look to find a family doctor. I was in the dark. So, I was quite pleased to receive that phone call; it took away some of the pressure and the stress of finding a family doctor.” [intervention patient 04]*

*“(…) the [navigator] (…) called me to tell me which doctor I had and on the day of my first appointment, it took a lot of stress out of me.” [intervention patient 04]*

e) Navigation is appreciated but unhelpful

The fifth sub-theme found is that the navigation intervention is appreciated, but unhelpful. Some SV patients considered the PWS positive, but did not need it because, for example, they were resourceful and autonomous or they were able to access care on their own. Some mentioned that the intervention did not change the way they navigate the healthcare system or the way they access information regarding the system:

*“Yes, the information was useful, but I don’t really see how I can put any of this into practice. I only count on having a doctor. But yes, the* [*navigator] was nice to me… I don’t consider that it changed how I access care”.* [*intervention patient 05]*

## Discussion

The purpose of this study was to describe and better understand the expectations and needs of SV patients in Montérégie (Québec, Canada) regarding navigation interventions for PHC services.

We identified three broad themes of expectations and needs of the SV population: communication expectations and needs (needs help to understand health and social service providers, expectations to successfully express oneself or to make oneself heard, needs to discuss the medical appointment, and expectations to help bridge the communication gap between patients and their PHC providers); relational expectations and needs regarding emotional and psychosocial support; and pragmatic expectations and needs (information on resources, being informed about the FMG (e.g., policies, resources, or providers), and physical support and accompaniment. These themes are consistent with the roles and definitions of patient navigators found in the literature, such as eight of the nine characteristics identified by Kelly et al. [15]: 1) advocacy; 2) care coordination (including transitions, discharge, and rehabilitation); 3) case monitoring and patient needs assessment; 4) community engagement; 5) education (including self-management and empowerment); 6) administration and research activities; 7) psychosocial support; and 8) navigation of services.

Among the sub-themes of the expectations and needs of SV patients reported in our results: expectation to help bridge the communication gap between patients and their PHC providers (communication expectations and needs), and physical support (pragmatic expectations and needs) are not typically addressed in navigation interventions [12, 15, 22]. Our results suggest, therefore, that it may be relevant to account for these expectations when designing navigation interventions. Typically, navigation interventions focus on SV populations [38], thus, it remains unclear whether the expectations and needs of SV populations regarding these interventions differ from those of the general patient population. Additionally, our results suggest that it may be relevant to ask SV patients about their needs and expectations when designing, or while providing a navigation intervention.

Regarding the communication expectations and needs theme, our findings suggest that SV patients need navigators to help them to understand health and social service providers. This is similar to previous studies reporting that patients expect navigators to explain what the doctor said [22]. Expectations to successfully express oneself or to make oneself heard, needs to discuss medical appointments, and expectation to help bridge the communication gap between patients and their PHC providers emerge from our study. Preliminary finding regarding appreciation of patients who received the PWS suggest that discussing appointments (e.g., navigator providing communication tips) is appreciated by the SV patients. This preliminary finding corroborates those of other studies reporting that patients appreciate the communication support provided by navigation interventions and the positive relationship between navigation interventions and communication between the patient and the medical team [16, 22, 39]. Thus, adding this feature to navigation interventions could be important.

Regarding relational expectations and needs, our findings suggest that SV patients expect the navigator to provide emotional and psychosocial support, namely reassurance. This is consistent with previous studies regarding participants’ expectations of navigation interventions [12, 22] and others that identified emotional support as an aspect of navigation interventions that patients appreciate [12, 23, 40, 41].

Regarding pragmatic expectations and needs, our results suggest that SV patients may expect navigation interventions to provide information on resources, including types, availability and location of resources (resources tailored to patients’ realities), and processes to follow to access and use these resources. They may also expect to be informed about the FMG (services provided, providers). Thus, our results suggest the navigator acts as a bridge between the health system and the biopsychosocial needs of SV patients. This is consistent with previous studies that found patients’ expectations of navigation interventions included assistance accessing information and solving problems, such as how to schedule appointments and where to go for medical tests [12, 16]. We note that the navigator is very important in navigation interventions because participants in our study consistently mentioned the navigator when they expressed their expectations and needs in relation to navigation interventions. Expectations and needs related to physical support emerge from our study.

Finally, preliminary findings from SV patients who received the PWS (findings will be reported elsewhere) suggest that they do not all find navigation interventions necessary, although many mentioned appreciating their interactions with the navigator. This corroborates research findings reported by others, such as Ploeg et al. [14], indicating that, despite appreciating navigation interventions, not all patients consider they need them.

### Strengths and limitations

To ensure credibility and transferability we recruited SV patients who received PWS and those who did not from three different FMGs. We made this choice because it allowed for triangulation of data [42] and diversification of patient perspectives regarding expectations and needs of navigation interventions, thereby allowing us to generate results to inform the design of future studies or navigation interventions. Member checking was not used in this study because as more research suggest that it may not improve study quality [43]. To further enhance transferability, we have provided a detailed description of the study participants and contexts [42] and the variation in the sample allowed for a wide variety of participant accounts. However, our participants live in a particular social context that is different from that of other Canadian’s provinces (Nova Soctia, British Columbia, Manitoba, New-Brunswick, Ontario, and Prince Edward Island) and countries in several fundamental aspects (population, socio-demographic characteristics, health care services, social support, and environmental factors) [44]. Thus, our results are likely not transferable in these contexts. The fact that some participants received the PWS may have influenced their expectations and needs regarding navigation interventions. This is why we provided a brief description of the intervention they received. The triangulation of data coming from patients who did not receive the PWS allowed us to ensure the consistency of themes found in both groups of patients. Finally, this study may have been affected by social desirability bias. As the study was conducted in collaboration with the FMGs where the family physicians of the interviewed patients were located, they may have deliberately omitted negative expectations or criticisms of the PWS or other services.

## Conclusion

Using a descriptive qualitative research approach, we found that SV patients’ have communication, relational and pragmatic expectations and needs regarding navigational interventions. Specifically, SV patients need support to understand health and social providers, to make themselves understood or heard by providers, to discuss medical appointments in advance, and to bridge the communication gap between themselves and their PHC providers. Their relational expectations are emotional and psychosocial support (responding to distress, providing comfort). Their pragmatic expectations relate to information on resources (types, locations, availability) and FMGs, and also physical support. Moreover, for navigation programs to be appreciated and helpful to SV patients, it may be relevant to take into account the expectations and needs reported here, in particular those pertaining to preparation for medical appointments, emotional support and provision of information on available resources, when designing and implementing them. Some SV patients may not need this navigation intervention at all, especially if they are resourceful and self-sufficient. Further studies are needed to explore the problems faced by SV patients receiving navigation interventions and to assess the effectiveness or appreciation of navigation interventions that take into account expectations and needs of patients from this sub-population group.

## Data Availability

As per the ethical approval received for this study and the consent provided by our participants, the datasets analysed during the current study are not publicly available given they contain information that could compromise participants’ privacy but are available from the corresponding author on reasonable request.

## Abbreviations

IMPACT: Innovative Models Promoting Access to Care Transformation
PHC: Primary healthcare
PWS: Patient welcome service
